# Veterinary Medical Association of New York County

**Published:** 1895-05

**Authors:** J. Elmer Ryder

**Affiliations:** Secretary


### VETERINARY MEDICAL ASSOCIATION OF NEW YORK COUNTY.

THE last regular meeting of the Veterinary Medical Association was held on Tuesday, April 2, 1895, at the New York College of Veterinary Surgeons, at 8 o'clock, with the President, Dr. Huidekoper, in the chair.

On roll-call the following members were present, viz.: Drs. Amling, Burns, C. C. Cattanach, J. J. Cattanach, J. S. Cattanach, Dickson, Delaney, Doyle, Ellis, Ferster, Field, Giffen, Gill, Glover, Huidekoper, Hanson, Loomes, Leighton, Lamkin, O'Shea, Ryder, Swan, Sherwood, Sherk, and Turner. Over fifty prominent horsemen and horseshoers were present as guests.

The minutes of the last meeting were read and approved.

The Board of Censors reported favorably the names of Drs. H. S. Field, C. E. Caulfield, H. Wellner, J. J. Foy.

It was moved and seconded that the report be accepted. Carried.

The Board of Censors also reported that they had laid the resignation of Dr. Liautard over until the next regular meeting.

A letter was read from Robert Bonner, Esq., expressing his regret that he could not attend.

The next order of business was the reading of papers:

Mr. David Roberge read a very interesting paper upon "Horseshoeing." (This paper will appear in the June number of the JOURNAL.)

Dr. Huidekoper followed with a paper upon the same subject, after which a general discussion took place, in which the majority of the members took part.

The next order of business was the election of new members.

It was moved and seconded that the by-laws be suspended and that the Secretary cast the ballot for all the applicants. Carried.

The Secretary cast a ballot declaring Drs. Foy, Field, C. Caulfield, and Wellner elected members of the Association.

On account of the lateness of the hour it was moved and seconded that all business be laid over until the next regular meeting. Carried.

The subject of horseshoeing will be continued at the April meeting.

Moved and seconded that the meeting adjourn. Carried.

J. ELMER RYDER,
Secretary.

